# Painful ophthalmoplegia in a patient with a history of marginal zone lymphoma

**DOI:** 10.1186/s41824-021-00113-2

**Published:** 2021-10-07

**Authors:** C. Van Bogaert, C. Mathey, I. Vierasu, N. Trotta, L. Rocq, A. Wolfromm, V. De Wilde, S. Goldman

**Affiliations:** 1 Department of Nuclear Medicine, CUB-Hôpital Erasme, Anderlecht, Belgium; 2 Department of anatomopatholgy, CUB-Hôpital Erasme, Anderlecht, Belgium; 3 Department of haematology, CUB-Hôpital Erasme, Anderlecht, Belgium

**Keywords:** Painful ophthalmoplegia, Lymphoma, Tolosa Hunt syndrome

## Abstract

A 73-year-old man with a history of marginal zone lymphoma was admitted to the emergency room for diplopia and ipsilateral headache. The Fluorine-18-fluorodeoxyglucose positron emission tomography/computed tomography (^18^F-FDG PET/CT) demonstrated intense and symmetrical hypermetabolism of the cavernous sinuses, and hypermetabolic lesions diffusely in the lymph nodes and bones. The diagnosis of high-grade relapse of lymphomatous disease was made. In this context, the homogenous and symmetric lesion of the cavernous sinuses, without any other encephalic or meningeal lesions, raised the hypothesis of a paraneoplastic origin. A plausible paraneoplastic link between the neuro-ophthalmological lesion and the malignant disorder is IgG4-related disease, a condition that may be associated with lymphoma. As in our case, this diagnosis is often presumptive because histopathological confirmation is difficult to obtain.

## Background

Painful ophthalmoplegia refers to periorbital or hemicranial pain associated with ipsilateral oculomotor nerve palsy. It may be caused by any process exerting a mass effect simultaneously on the internal carotid artery and the cavernous sinus and/or the superior orbital fissure. It comprises several etiologies, including neoplastic, vascular, inflammatory, or infectious disorders (Gladstone 2007).

In the presence of painful ophthalmoplegia, the Tolosa Hunt syndrome (THS), firstly described in 1954, is a diagnosis of exclusion. It is characterized by a unilateral headache associated with a paralysis of the ipsilateral third, fourth, and/or sixth cranial nerves caused by nonspecific granulomatous inflammation of the cavernous sinus, the superior orbital fissure, or the orbit (generally termed orbital pseudotumor). This inflammatory process is of unknown etiology and rapidly resolving after corticoid therapy (Gladstone 2007; Lueck 2018). Interestingly, it has been reported in IgG4-related disease (IgG4-RD), a multi-organ immune-mediated fibro-inflammatory condition that may be associated with lymphoma (Carbone et al. 2015; Lindfield et al. 2012; Wang et al. 2020).

## Case presentation

A 73-year-old man was admitted to the emergency room for diplopia and ipsilateral headache for about ten days. He mentioned a weight loss of seven kilos over the last few months, attributed to a loss of appetite. Three months before admission, he had surgery for hemorrhoid repair after rectal bleeding, leading to a discontinuation of the anticoagulation required for chronic auricular fibrillation. As other antecedents, we retained ischemic cardiomyopathy, arterial hypertension, and marginal zone lymphoma (MZL) treated by splenectomy in 2012.

At the admission, the patient had completely normal parameters with a blood pressure of 120/60 mmHg, heart rate of 65 bpm, temperature of 36.3°C, and oxygen saturation in ambient air of 95%. The clinical examination revealed a systolic heart murmur irradiating in both carotids. Neurological examination showed oculomotor paralysis of the left eye with ptosis, unreactive mydriasis, and divergent strabismus. Blood tests only showed severe hyperleukocytosis (with 95% of lymphocytes) and signs of cytolysis. The brain CT was unremarkable.

In the context of a discontinued anticoagulation, an ischemic stroke of cardio-embolic origin located in the left mesencephalic region was suspected and the patient was admitted to the stroke unit.

## Investigations

Among the additional biological dosages made during the hospitalization (hemostasis work-up, lipid profile, protein electrophoresis, lymphocyte typing, and immunological workup including immunoglobulins, ANF, and ANCA), lymphocyte typing revealed a pathological population of B lymphocytes.

Brain magnetic resonance imaging (MRI) demonstrated no recent ischemic lesion and no brain mass.

**Fig. 1:**
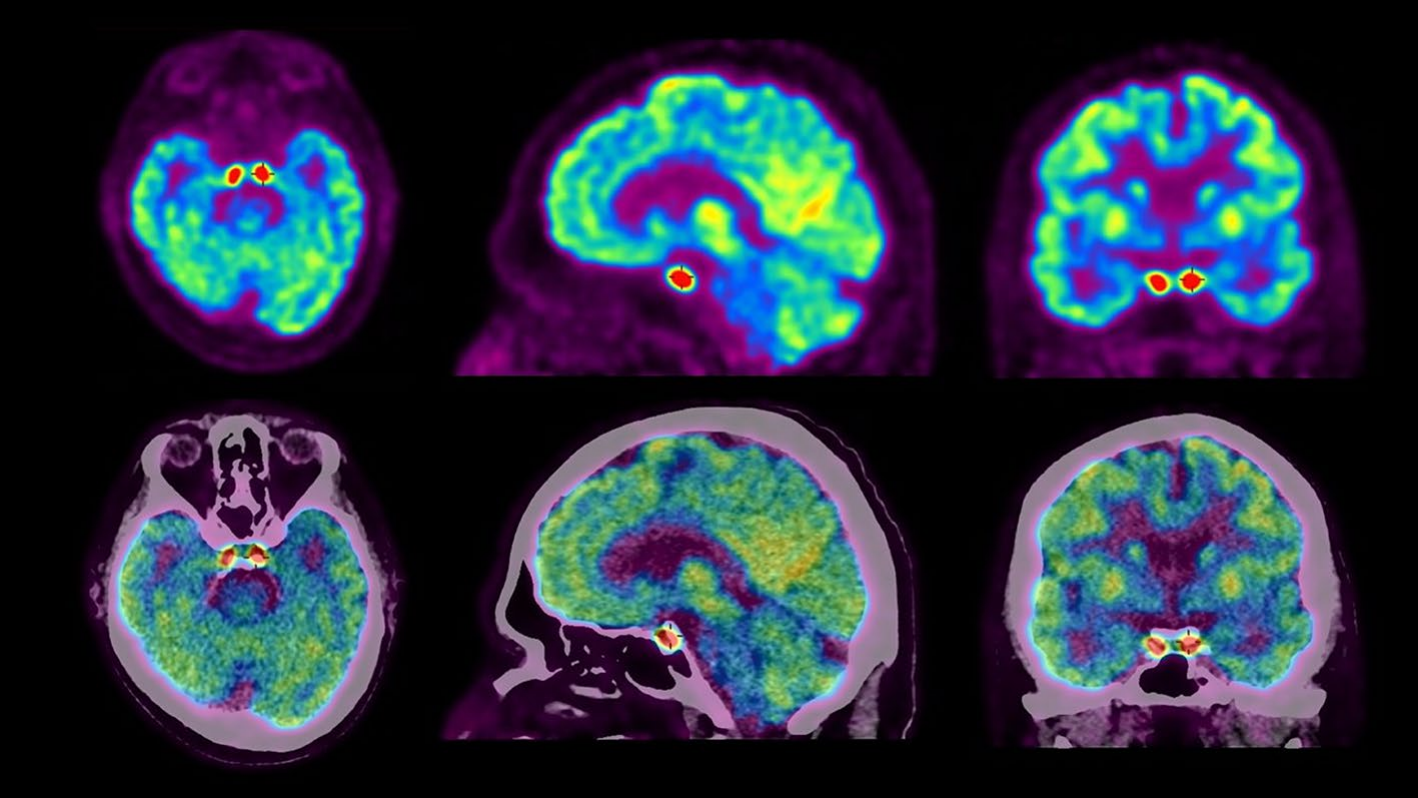
Cerebral ^18^F-FDG PET/CT demonstrating the presence of intense and symmetrical hypermetabolism of the cavernous sinuses

**Fig. 2:**
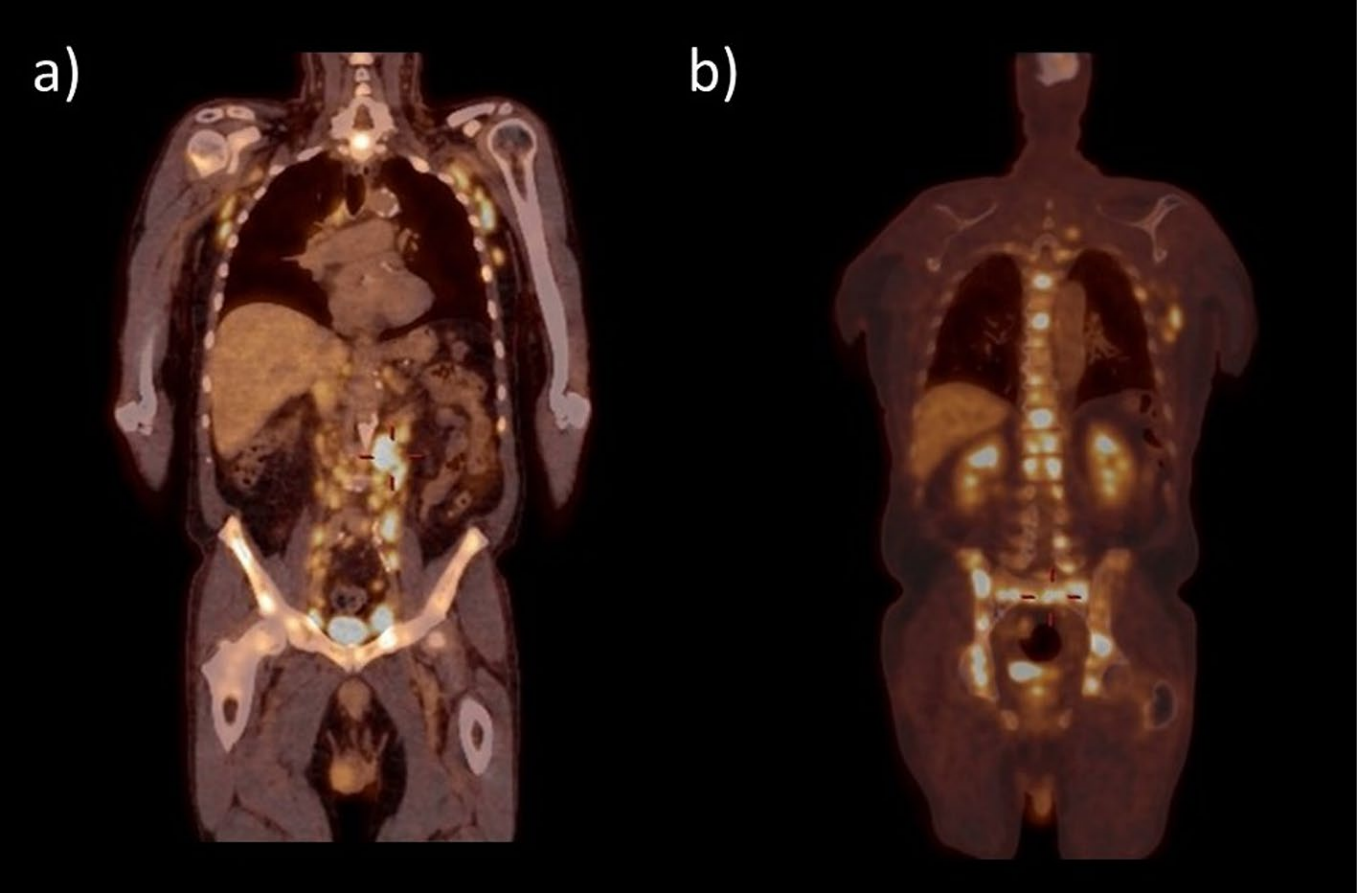
Whole body ^18^F-FDG PET/CT demonstrating hypermetabolic lesions diffusely in the lymph nodes (**a**) and bones (**b**), highly suggestive of a high-grade relapse of the lymphomatous disease

Cerebral and whole-body Fluorine-18-fluorodeoxyglucose positron emission tomography/computed tomography (^18^F-FDG PET/CT) demonstrated intense and symmetrical hypermetabolism of the cavernous sinuses (Fig. 1), associated with hypermetabolic lesions diffusely in the lymph nodes and the bones, highly suggestive of a high-grade relapse of the lymphomatous disease (Fig. 2).

The cerebrospinal fluid examination was unremarkable (proteins: 38 mg/dL; 1 leucocyte/mm^3^ and 3 erythrocytes/mm^3^).

Bone marrow and right axillary lymph node sampling were indicative of a diffuse large B-cell lymphoma (DLBCL).

Transformation of a MZL to a DLBCL was then concluded.

Concerning the neuro-ophthalmological symptoms, a local homogenous and symmetrical lymphomatous infiltration of the cavernous sinuses without any other sign of encephalic or meningeal lesions seemed unlikely. Therefore, we raised the hypothesis of an IgG4-RD. Indeed, IgG4-RD may cause an orbital inflammatory disease and an etiological link is suspected between IgG4-RD and lymphoma (Lindfield et al. 2012; Wang et al. 2020; Bledsoe et al. 2018; Takahashi et al. 2009; Peng et al. 2020; Wallace et al. 2016; Yamamoto et al. 2012). Serological dosage of IgG4 was therefore performed and returned negative.

## Treatment

**Fig. 3:**
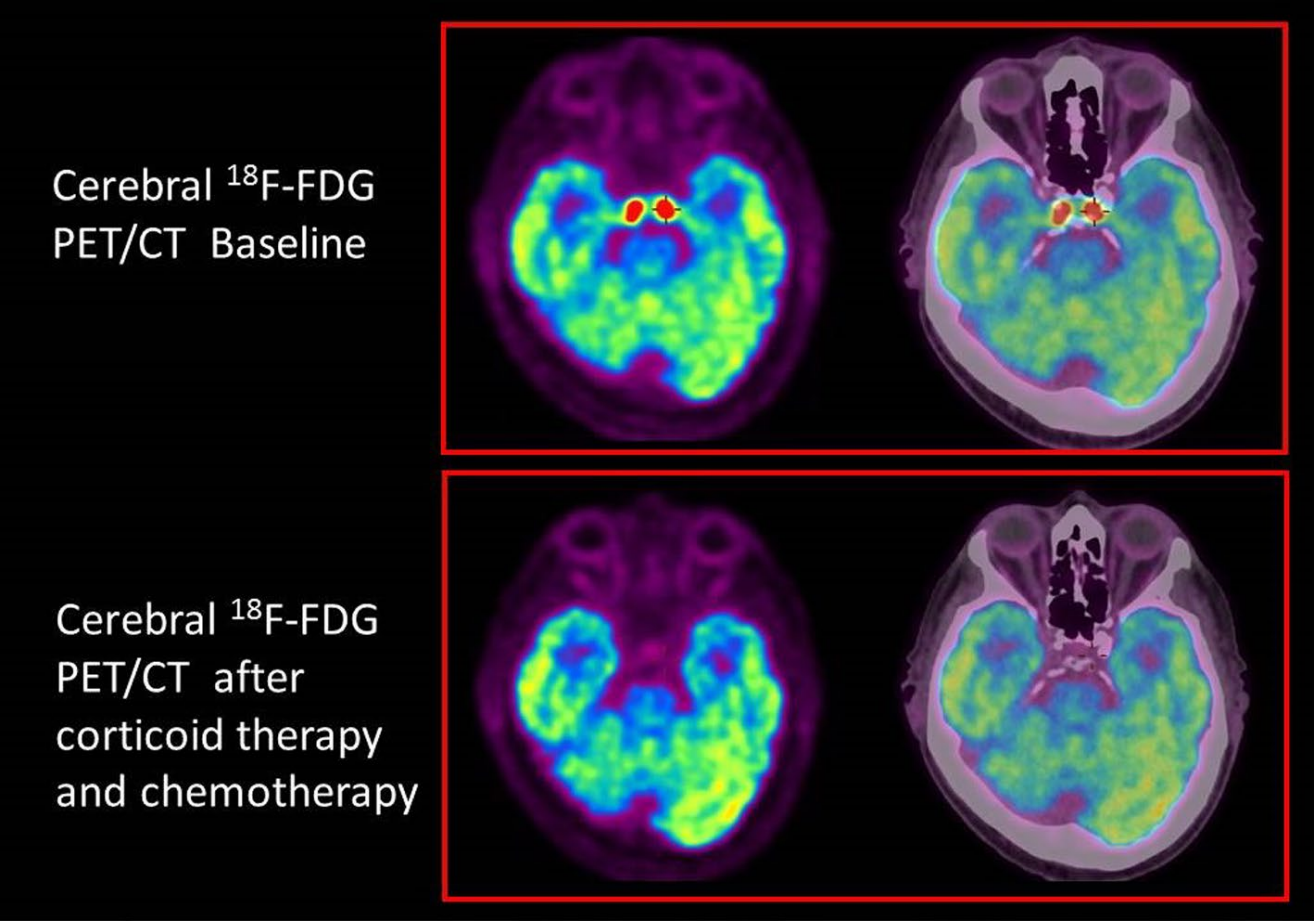
Cerebral ^18^F-FDG PET/CT demonstrating a complete response after corticoid therapy, 6 cycles of R-CHOP and 4 cycles of HD-MTX

**Fig. 4:**
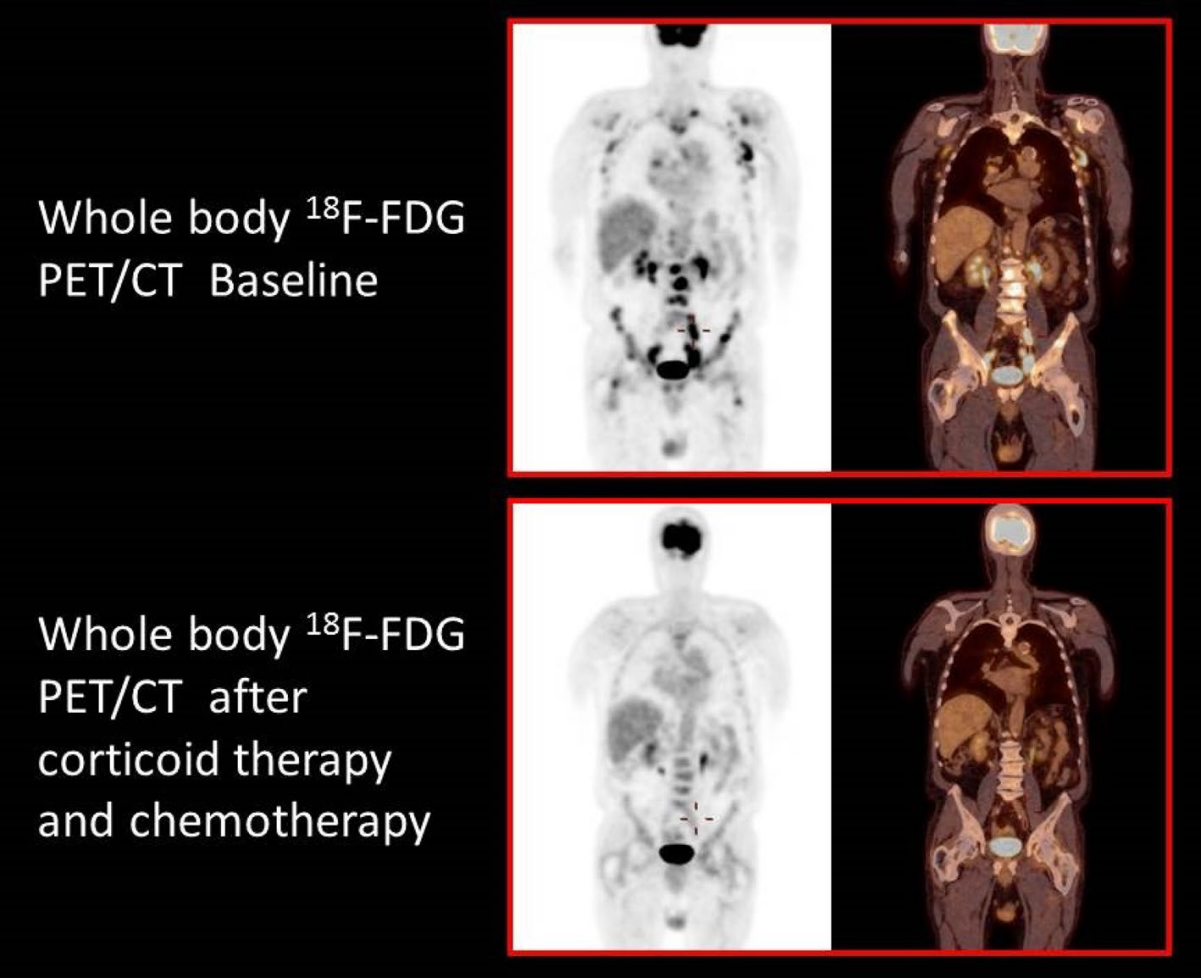
Whole body ^18^F-FDG PET/CT demonstrating a complete response after corticoid therapy, 6 cycles of R-CHOP, and 4 cycles of HD-MTX

The patient was treated by corticoid therapy with rapid and complete resolution of the neuro-ophthalmologic symptoms. The cerebral (Fig. 3) and whole body (Fig. 4) ^18^F-FDG PET/CT demonstrated a complete response after 6 cycles of Rituximab with Cyclophosphamide, Doxorubicin, Vincristine and Prednisone (R-CHOP) and 4 cycles of high-dose methotrexate (HD-MTX).

## Outcome and follow-up

Six months later, the patient presented a DLBCL recurrence in the supra- and sub-diaphragmatic lymph nodes with no neurological symptom. He was treated by Rituximab- with Gemcitabine/-, Dexamethasone, and Cisplatine (R-GDP), shifted to Rituximab with Gemcitabine, Dexamethasone, and Carboplatin (R-GDCarbo) for nephrotoxicity, with complete response after 3 cycles.

Unfortunately, before the end of this therapy, the patient died from a septic shock accompanying a SARS-CoV-2 infection (COVID-19).

## Discussion

THS is a painful ophthalmoplegia related to the so-called orbital pseudotumor (also called idiopathic orbital inflammatory disease), a nonspecific granulomatous inflammation of the orbit, the superior orbital fissure, or the cavernous sinuses (Gladstone 2007; Lueck 2018; Wasmeier et al. 2002). MRI may help in the diagnosis, but it can be unremarkable in one-third of the cases (Gladstone 2007). Biopsy is an invasive examination and should only be considered for the management of patients with rapidly progressive neurologic impairment.

The case here reported illustrates the value of cerebral ^18^F-FDG PET/CT images since this examination provided a clear demonstration of a homogenous and symmetric involvement of the cavernous sinuses that had not been seen on the CT and MRI.

Without any other encephalic or meningeal lesions, this cavernous sinus involvement could not be reasonably attributed to an isolated local lymphomatous invasion. This was support by the rapid clinical response to corticoid therapy, as expected in THS. Therefore this neuro-ophthalmological manifestation had most likely a paraneoplastic origin. Since paraneoplastic syndromes are generally related to autoimmune processes, we considered the hypothesis that THS in this case was a manifestation of a lymphoma-induced IgG4-RD. Indeed, on the one hand, THS has been described in association with IgG4-RD (Lindfield et al. 2012). On the other hand, evidence is accumulating that this chronic inflammatory state is associated with high incidence to lymphoma (Wang et al. 2020). In particular, an association has been reported between DLBCL and IgG4-RD, notably with autoimmune pancreatitis or orbital inflammation (Wang et al. 2020; Takahashi et al. 2009; Peng et al. 2020). In the majority of reported cases, IgG4-RD occurred precedently or concurrently to the development of malignant lymphoma. However, some studies showed that patients with a history of malignancy—including lymphoma—are predisposed to develop IgG4-RD compared to the general population (Wallace et al. 2016; Yamamoto et al. 2012). A strong etiological link between IgG4-RD and lymphoma has therefore been proposed in the literature (Lindfield et al. 2012; Wang et al. 2020; Bledsoe et al. 2018; Takahashi et al. 2009; Peng et al. 2020; Wallace et al. 2016; Yamamoto et al. 2012).

A diagnosis of IgG4-RD is often difficult to establish. Wallace et al. have demonstrated that only 51% of patients with active disease have elevated serum IgG4 concentrations (Wallace et al. 2016). In our case with negative serology, an invasive biopsy of the cavernous sinus to search for infiltration by IgG4 positive plasma cells was considered unjustified in light of the favorable evolution under treatment. Therefore, our diagnosis of IgG4-RD developed as a paraneoplastic syndrome related to DLBCL remains hypothetical.

## Conclusions

THS is a diagnosis of exclusion in the presence of painful ophthalmoplegia. It is characterized by a local nonspecific granulomatous inflammation.

Painful ophthalmoplegia requires investigation by MRI to exclude a local mass, but this method may not demonstrate and localize all inflammatory lesions in this clinical setting.

As shown in our patient, ^18^F-FDG PET/CT may be valuable in the work-up of painful ophthalmoplegia. Its high sensitivity helps to localize the causing lesions, including when the origin is an inflammatory process.

Considering the reported association of THS with malignancies, ^18^F-FDG PET/CT has a dual advantage in this context. First, it helps detect and characterize a local pathological process producing the syndrome. Second, it may potentially reveal a malignant disorder at the origin of the painful ophthalmoplegia.

Since the link between malignancy and painful ophthalmoplegia may be an IgG4-RD, other manifestations of this disorder may also become apparent on the ^18^F-FDG PET/CT performed.

## Data Availability

Not applicable.

## Abbreviations

CT: computed tomography
^18^F: fluorine-18
FDG: fluorodeoxyglucose
DLBCL: diffuse large B-cell lymphoma
HD-MTX: high-dose methotrexate
IgG4-RD: IgG4-related disease
MALT: mucosa-associated lymphoid tissue
MRI: magnetic resonance imaging
MZL: marginal zone lymphoma
PET: positron emission tomography
R-CHOP: rituximab-cyclophosphamide/doxorubicin/vincristine/prednisone
R-GDCarbo: rituximab-gemcitabine/dexamethasone/carboplatin
R-GDP: rituximab-gemcitabine/dexamethasone/cisplatine
THS: Tolosa Hunt syndrome

## References

[CR1] Bledsoe JR, Wallace ZS, Stone JH, Deshpande V, Ferry JA (2018). Lymphomas in IgG4-related disease: clinicopathologic features in a Western population. Virchows Arch Int J Pathol.

[CR2] Carbone T, Azêdo Montes R, Andrade B, Lanzieri P, Mocarzel L (2015). Orbital pseudotumor: uncommon initial presentation of IgG4-related disease. Case Rep Rheumatol.

[CR3] Gladstone JP (2007). An approach to the patient with painful ophthalmoplegia, with a focus on Tolosa-Hunt syndrome. Curr Pain Headache Rep.

[CR4] Lindfield D, Attfield K, McElvanney A (2012). Systemic immunoglobulin G4 (IgG4) disease and idiopathic orbital inflammation; removing ‘idiopathic’ from the nomenclature?. Eye.

[CR5] Lueck CJ (2018). Time to retire the Tolosa-Hunt syndrome?. Pract Neurol.

[CR6] Peng X, Jing H, He W (2020). Bilateral IgG4-related ophthalmic disease with diffuse large B-cell lymphoma of the right eye: a case report. Ophthal Plast Reconstr Surg.

[CR7] Takahashi N, Ghazale AH, Smyrk TC, Mandrekar JN, Chari ST (2009). Possible association between IgG4-associated systemic disease with or without autoimmune pancreatitis and non-Hodgkin lymphoma. Pancreas.

[CR8] Wallace ZS, Wallace CJ, Lu N, Choi HK, Stone JH (2016). Association of IgG4-related disease with history of malignancy. Arthritis Rheumatol.

[CR9] Wang H, Su T, Kang L, Yang L, Wang S (2020). Diffuse large B cell lymphoma in a preceding IgG4-related disease with kidney restricted lambda light chain expression: case report and literature review. BMC Nephrol.

[CR10] Wasmeier C, Pfadenhauer K, Rösler A (2002). Idiopathic inflammatory pseudotumor of the orbit and Tolosa-Hunt syndrome: are they the same disease?. J Neurol.

[CR11] Yamamoto M, Takahashi H, Tabeya T, Suzuki C, Naishiro Y, Ishigami K (2012). Risk of malignancies in IgG4-related disease. Mod Rheumatol.

